# Extra-pair paternity in the long-tailed finch *Poephila acuticauda*

**DOI:** 10.7717/peerj.1550

**Published:** 2016-01-05

**Authors:** Erica P. van Rooij, Lee A. Rollins, Clare E. Holleley, Simon C. Griffith

**Affiliations:** 1 Department of Biological Sciences, Macquarie University, Australia; 2 Centre for Integrative Ecology, Deakin University, Geelong, VIC, Australia; 3 Institute for Applied Ecology, University of Canberra, Canberra, ACT, Australia

**Keywords:** Extra-pair paternity, Sexual selection, Polyandry, Infidelity, Social monogamy, Estrildid

## Abstract

Although the majority of passerine birds are socially monogamous, true genetic monogamy is rare, with extra-pair paternity (EPP) occurring in almost 90% of surveyed socially monogamous species. We present the first molecular data on the genetic breeding system of the long-tailed finch, *Poephila acuticauda*, a grass finch endemic to the tropical northern savannah of Australia. Although the species forms socially monogamous pair bonds during the breeding season, we found that extra-pair males sired 12.8% of 391 offspring, in 25.7% of 101 broods. Our findings provide only the second estimate of extra-pair paternity in the estrildid finch family.

## Introduction

The majority of passerine birds are socially monogamous (Cockburn, 2006), and yet extra-pair paternity (EPP) was found to be present in over 90% of species for which paternity had been investigated when reviewed by Griffith, Thuman & Owens (2002). In socially monogamous species with long-term pair bonds, both males and females are constrained in their choice of social mates by the low availability of unpaired individuals, and extra-pair mating may enable females to gain genetic benefits for at least some of their offspring (Akcay & Roughgarden, 2007; Griffith & Immler, 2009), or to secure against the infertility of a male partner (Sheldon, 1994; Griffith, 2007). In addition, focus has recently started shifting towards a number of non-adaptive models that can explain the incidence of the fairly widespread extra-pair paternity observed in many species of bird (Forstmeier et al., 2014).

An improved understanding of extra-pair paternity in birds will depend partly on further experimental work on well studied and amenable research species. For example, there are a handful of European and North American species that have already been the focus of dozens of studies of extra-pair paternity across multiple populations (e.g., blue tit *Cyanistes caeruleus* (e.g., Foerster et al., 2003); house sparrow *Passer domesticus* (Griffith et al., 1999); collared flycatcher *Ficedula albicollis* (Veen et al., 2001)). The alternative route to new insight will likely come from further comparative studies and meta-analyses that make use of the expanding dataset on variation in extra-pair paternity across species (e.g., Cleasby & Nakagawa, 2012; Griffith, Thuman & Owens, 2002). Even in the passerines, the most extensively studied avian family, many sub-families have yet to be studied at all or have been investigated through just one or two species. To date, our understanding of extra-pair paternity in the passerine sub-family Estrildidae is based only on the study of the Australian zebra finch *Taeniopygia guttata* which has been the focus of two separate estimates of extra-pair paternity in the wild (Birkhead et al., 1990; Griffith et al., 2010), and numerous studies in captivity (Forstmeier et al., 2011; Baran & Adkins-regan, 2014).

The long-tailed finch *Poephila acuticauda* is a close relative of the zebra finch and has a similar social mating system to that species, with evidence of long-term partnerships between males and females that last across multiple reproductive attempts within and across breeding seasons (zebra finch see Zann, 1996; long-tailed finch see Van Rooij & Griffith, 2011; Van Rooij & Griffith, 2013). Here we report on a study characterizing the occurrence of extra-pair paternity in a single population of the long-tailed finch near the town of Wyndham in Western Australia. Our work is completely exploratory and we had no particular expectation of the likely level of genetic polyandry in this species, and nor were we testing any hypothesis regarding the distribution across individuals. Our study characterises the incidence of genetic polyandry by genotyping parents and offspring using microsatellite genotyping—a method that has remained the best practice for over fifteen years (Griffith, Thuman & Owens, 2002).

## Methods

### Study area, species and general methods

The long-tailed finch is a common Australian grass finch of the family Estrildidae, and endemic to Northern Australia. Pairs are highly sedentary and remain in the same area during the breeding season and across years, with two or three nesting attempts per breeding season (Van Rooij & Griffith, 2011). Both members of the pair participate in nest construction, incubation, brooding and feeding of the altricial young (Van Rooij & Griffith, 2011; Van Rooij & Griffith, 2013). This work focused on the western sub-species *Poephila acuticauda acuticauda* in an area to the west of the putative contact zone with the other sub-species *P. a. hecki* (Jennings & Edwards, 2005; Rollins et al., 2012). Fieldwork was conducted during three breeding seasons (March–September) in three years (2008–10) near Wyndham, Western Australia (S15°33′38″, E128°08′59″). The nominate sub-species occurs in this area, which is. The study area consisted of 108 ha of savannah woodland with Eucalypt trees providing natural cavities for nesting as well as artificial nest boxes supplied to facilitate this study and work on the Gouldian finch *Erythrura gouldiae* at the same site (Brazill-Boast et al., 2011).

We sampled 101 complete families (all offspring and both putative parents) over three breeding seasons (24 in 2008, 62 in 2009, 15 in 2010). All nest boxes were checked for new nesting attempts every six days. Active nests were checked daily from two days before the expected hatching date (12 days from the onset of incubation; Higgins, Peter & Cowling, 2006). At the age of twelve days all nestlings were banded, measured and a small blood sample was taken (and stored in ethanol). Adults were captured and banded using mist-nets at watering points (in creek beds) or with hand-nets when they entered nests. For each adult at the time of first capture we took a small blood sample (<20 µl) from the brachial vein and stored in ethanol for later molecular work. All adults were banded with a numbered aluminium band (supplied by the Australian Bird and Bat Banding Scheme, ABBBS) and a unique combination of three colour bands. Putative parents were either captured while feeding nestlings or confirmed by direct observation of colour-banded parents visiting the nest to feed the offspring (details in Van Rooij & Griffith, 2013). Adults were sexed using molecular sex markers as part of an earlier study (Van Rooij & Griffith, 2010). This work was approved under the authority of a Macquarie University Animal Ethics Committee Authorisation #2007/038, and the Western Australia, Department of Environment and Conservation (no. BB 002563).

### Molecular analyses of paternity

DNA was extracted from blood samples using the Puregene DNA Purification Kit (Qiagen). We used five fluorescently labeled microsatellite loci (*Tgu*1, *Tgu* 3, *Tgu*4, *Tgu*8 and *Tgu*12) that had previously been isolated and characterised in the closely related zebra finch (Forstmeier et al., 2007). The details of the characterization of the loci in this species are given in Table 1. All samples were run in two multiplex PCR reactions using a Qiagen Multiplex Kit at one-fifth the recommended volume, using multiplex PCRs. Samples were genotyped on a 48-Capillary 3730 DNA Analyser (Applied Biosystems, Foster City, CA, USA) using GS-500 (Liz) in each capillary as a size standard. Allele sizes were estimated on GeneMapper version 3.7 (Applied Biosystems 2004). Combined non-exclusion probabilities were calculated by CERVUS 3.0 (Kalinowski, Taper & Marshall, 2007).

**Table 1:** Characteristics of the molecluar markers used in the study. Allele size ranges, number of alleles, the level of heterozygosity, the probability of genotype sharing, probability of false inclusion, deviation from Hardy–Weinberg equilibrium and null allele frequencies, all based on the allele frequencies detected in 112 individuals (53 females, 59 males), which bred in the study area in 2008–2010.

Locus	Allele size range	Number of alleles found	Hetero-zygosity^a^	Probability of genotype sharing^b^	Probability of false inclusion^c^	Dev. from HW	Null allele frequency estimate
*Tgu*1	170–192	10	0.90	1.9 × 10^−2^	0.19	Yes	0.0097
*Tgu*3	144–190	18	0.95	5.2 × 10^−3^	0.10	No	0.0679
*Tgu*4	99–147	19	0.96	3.3 × 10^−3^	0.08	No	0.2212
*Tgu*8	193–239	19	0.95	4.5 × 10^−3^	0.10	Yes	0.0417
*Tgu*12	101–139	16	0.94	7.7 × 10^−3^	0.12	No	−0.0069

**Notes.**

a Heterozygosity was calculated as (1 − *q*), where *q* is the mean allele frequency derived from Cervus (following Kalinowski, Taper & Marshall, 2007).

b For a single locus the probability that two unrelated individuals will share the same genotype is given by *q*^2^(2 − *q*) (Kalinowski, Taper & Marshall, 2007), where *q* is the mean allele frequency (following Kalinowski, Taper & Marshall, 2007).

c For a single locus the probability of false paternal inclusion is given as 2*q* − *q*^2^ (Kalinowski, Taper & Marshall, 2007).

HW Hardy–Weinberg Equilibrium Null allele frequency estimate calculated in CERVUS

We assessed the occurrence of EPP by comparing offspring with both putative parents across the microsatellite loci. Most offspring and putative parents (*n* = 505) were successfully scored at all five microsatellite loci, but 19 (<5%) of the 393 offspring were scored at only four loci, because one locus failed to amplify. In the 112 adults genotyped, the loci were all highly variable ranging from 10 to 19 alleles per locus (Table 1; combined non-exclusion probabilities for this set of markers in this population were 0.012 for the first parent and 0.001 for the second parent). We used CERVUS 3.0 to assign maternity and paternity, then confirmed the output by manually comparing allele matching across loci in families. The mother was confirmed first and then maternal alleles were excluded when searching for the father. This approach combined the likelihood estimates (from CERVUS) with the more conservative traditional method of matching the inheritance of alleles at the co-dominant microsatellite loci. When social parents matched the offspring at four or more loci, in all cases, both methods concurred and we are confident of our assignment to social parents (or exclusion of social parents). In cases in which a social male was excluded, we searched the genotypes of all sampled males in the population to identify extra-pair sires. We again used CERVUS to perform a search of all males and a likelihood of assignment, and again these were compared manually for matching. In most cases the male identified as most likely by CERVUS was not considered to be the actual father on the basis of multiple mismatching loci. Paternity of extra-pair offspring was only assigned to an extra-pair male when at least four loci matched at the non-maternal allele.

## Results

In total, 393 offspring from 101 broods were genotyped with both their putative parents. 391 of the offspring matched with at least one of the maternal alleles at all of the loci scored. Eighty eight offspring had a mismatch with the social mother at a single maternal locus and five offspring mismatched with their social mother at two loci, but in these cases the mismatches were consistent with the presence of a null allele. i.e., the parent and/or offspring were scored as homozygotes, and most of these mismatches occurred at *Tgu*4 which was characterized as having a particularly high rate of null alleles. A single offspring in each of two broods mismatched with both the social mother and social father at two or more loci and these offspring were attributed to either intraspecific brood parasitism or eggs that were already in nests that were taken over by the social pair (i.e., 2 of 393, 0.5%). Mismatches between offspring and the social male occurred at a higher level (111 mismatched at one locus, 20 mismatched at two loci and 48 mismatched at three or more loci). Again, all of those with a single mismatch, and most all of those with two mismatches, could be attributed to the presence of null alleles. However, 50 of the 391 offspring (12.8%) that belonged to the social mother at a nest were determined to have been sired by a male other than the social male at the nest, on the basis that mismatches could not be explained by the presence of null alleles. These offspring matched the social mother and we conclude that they must therefore have been the result of extra-pair paternity, and they were distributed in 26 of 101 broods (25.7%). The rates were broadly similar across the three different years (see Table 2). Of the 26 nests that contained extra-pair offspring, nine (35%) contained a single chick sired by the extra-pair male, but in 13 nests, more than half the offspring were sired by the extra-pair male with one nest containing four nestlings, none of whom were sired by the social male.

**Table 2:** The extra-pair paternity across years in the study population. The incidence of extra-pair paternity (EPP) in a long-tailed finch population near Wyndham, WA in 2008, 2009 and 2010. The two cases of IBP have been excluded from the total sample below.

Year	No. of broods	No. of broods with EPP	% broods with EP nestlings	No. of nestlings	No. of EP nestlings	% of EP nestlings
2008	24	6	25.0%	91	10	11.0%
2009	62	16	25.8%	244	31	12.7%
2010	15	4	26.7%	56	9	16.1%
**Total**	**101**	**26**	**25.7%**	**391**	**50**	**12.8%**

Our sample of 101 broods was produced by 59 different females, with 31 females contributing a single brood to the sample, 18 sampled across two broods, seven with three, two with four and one female sampled across five broods. The incidence of extra-pair paternity did not seem to be driven by a few individual females or males, and indeed unique pairs were sampled multiple times and may have had extra-pair paternity in one of their broods but not others (full data provided in Table S1). For individuals that were measured multiple times there did not seem to be any pattern in the incidence of extra-pair paternity over time i.e., it does not appear more likely to occur in earlier or later broods either within a year, or across years (Tables S2 and S3). For example, 23 females bred multiple times within a season, and of these 10 had extra-pair offspring in neither brood; seven had extra-pair offspring in the first but not the second; five had extra-pair offspring in the second but not the first and one had extra-pair offspring in both broods (Table S2A). It is the same kind of pattern for males breeding multiply within a season (Table S2B), and males and females breeding across two consecutive seasons (Tables S3A and S3B). Furthermore, males and females that were sampled across multiple broods were more likely to have detectable polyandry (e.g., 7 out of 31 females with one brood were polyandrous, whereas 16 out of 28 females sampled in 2 or more broods were polyandrous in at least one of their broods). However, this is as expected if there was a random distribution of polyandry across all broods—the more broods you have, the more likely you are to have polyandry in at least one of them.

An extra-pair sire was identified for just 12 of the 50 extra-pair offspring from seven of the 26 broods sired by multiple males. In five of these broods, all extra-pair offspring shared the same father, but in the other two broods, the identified extra-pair sire did not father all of the extra-pair offspring. In Table 3 we have summarized the gains and losses made by the seven males that sired extra-pair offspring in relation to additional offspring sired but also the incidence of extra-pair paternity in their own social nests. Three of the seven extra-pair sires were cuckolded themselves and overall two had fewer genetic progeny as a result of extra-pair paternity. The other five males increased their genetic output by 20–67% through the siring of additional offspring outside the pairbond.

**Table 3:** The paternity gains and losses made by extra-pair sires. The gains and losses made by the seven males that were identified as extra-pair sires, both in offspring sired and offspring lost to paternity with their own social partner. The numbers reflect all of the reproductive effort that was detected and measured in the year in question.

**Male ID**	**Year gained EPP**	**EPO gained**	**Total offspring in social nest**	**No. offspring lost to EPP**	**Sum genetic offspring**	**Prop. diff. with EPP**
61384	2008	2	2	1	3	1.50
44885	2009	2	3	0	5	1.67
61109	2009	1	5	0	6	1.20
61315	2009	1	7	2	6	0.85
61316	2009	2	15	3	14	0.93
61332	2009	2	5	0	7	1.40
61566	2009	2	5	0	7	1.40

## Discussion

Like so many other species of bird, and particular like many other passerines, we found that social monogamy does not always equate with genetic monogamy in the long-tailed finch. In the population that we studied here, we detected a level of extra-pair paternity (13% of offspring in 26% of broods) that is very close to the average of 11% offspring in 19% of broods reported across all socially monogamous birds that were reviewed by Griffith, Thuman & Owens (2002). This finding contrasts with the only other estrildid finch that has been examined to date, the zebra finch, in which only about 2% offspring were found to be sired by extra-pair sires in two different wild populations (Birkhead et al., 1990; Griffith et al., 2010). We are confident that our overall sample size (101 broods from over 50 females), and molecular analysis provides a reasonable estimate of the level of extra-pair paternity in this species and population. However, because we did not systematically measure all of the morphological traits in adults in each of the years of study, and because our sample sizes in each year were relatively small, we were unable to explore the underlying determinants of variation in the level of extra-pair paternity across either females or males. We certainly encourage future studies of this, and other species, to accurately measure individual variation in morphological traits that may provide insight into possible selection through genetic polyandry on signals of age, attractiveness or quality.

Although we have not investigated the determinants of individual success at defending or gaining paternity, we can make some deductions about possible selection on extra-pair paternity from its incidence across individuals. For example, there is no clear pattern with respect to the incidence across a season or across years and this suggests that extra-pair paternity is not related to breeding experience or age. Individuals that had extra-pair offspring in one brood were not subsequently more likely than others to have extra-pair offspring in a subsequent brood. This suggests that there are not individual characteristics that dispose an individual to being repeatably genetically polyandrous over time.

We were unfortunately able to identify the fathers of only very few of the extra-pair offspring that we detected. This presumably reflects the fact that even though we sampled most of the birds breeding in the habitat patch in which we were working, adults appeared to range quite far out of the nesting habitat to forage, and presumably encountered many unsampled males in the broader area. The analyses and conclusions that we can draw from the few extra-pair sires that we did identify are limited. We found that all of them also had a partner with whom they bred in the same year in which they gained paternity. Although it is possible that many of the other unidentified extra-pair sires were not breeding, which may have been why they were not captured and blood sampled. Nevertheless, for the seven males that we did identify, three of them lost paternity in the same year that they gained extra-pair paternity, and two actually lost more offspring in their own nest than they gained through their infidelity. These observations are consistent with the idea that extra-pair paternity in this population is not strongly related to male quality. In some other passerine species in which there are indications that extra-pair paternity is related to male quality, it tends to be the case that the males that gain extra-pair offspring also tend to not lose paternity in their own nests (Whittingham & Dunn, 1999).

A recent study has identified small but significant differences in the sperm morphology of the two sub-species of the long-tailed finch, even though these are believed to have diverged only about 0.3 mya (Rowe et al., 2015). Given these differences, it is possible that genetic polyandry and sperm competition are involved in post-copulatory species isolation mechanisms, and that the function of extra-pair paternity is most important in the contact zone where the sub-species interact (Griffith, 2010). A laboratory study of another estrildid finch did find some evidence for post-copulatory fertilisation biases across two genetically incompatible morphs (Pryke, Rollins & Griffith, 2010). If such a process was present in the long-tailed finch, then it may mean that there was no adaptive function to genetic polyandry outside of the contact zone (in the area where we did this work), but may occur because of the potential value of such behaviour in the contact zone (which is relatively close). The fact that we have now observed some genetic polyandry in this species suggests that this idea may be worthy of further attention.

In summary, our results provide the first estimate of the level of extra-pair paternity in this socially monogamous species, and just the second in the family of estrildid finches. The level of extra-pair paternity in this species is around the average level seen across all socially monogamous birds. The incidence of extra-pair paternity across individuals in the population does not appear to be consistently biased towards any individuals and appears to be neither more or less likely to occur in successive broods belonging to either individual males or females.

## Supplemental Information

10.7717/peerj.1550/supp-1 Table S1 Data on the incidence of extra-pair offspring across all nests Data on the incidence of extra-pair offspring in 101 nests across the three years sorted by female ID and date order of nests (EPO, extra-pair offspring; WPO, within-pair offspring; Prop EP, proportion of offspring that are sired by extra-pair male).

10.7717/peerj.1550/supp-2 Table S2 Data on the incidence of extra-pair paternity across successive broods for parents that bred multiple times within a year The proportion of extra-pair offspring in the successive broods in the (A) females and (B) males that bred multiple times within the same year (broods arranged in date order). Signs denote the direction of change over time: n/c, no change; +, increase; −, decrease.

10.7717/peerj.1550/supp-3 Table S3 The level of extra-pair paternity across broods for parents that bred multiple times across different years. The proportion of extra-pair offspring in the successive broods in the (A) females and (B) males that bred across two different years (the values are from their broods that are last in year 1 and earliest in year 2). Signs denote the direction of change over time: n/c, no change; +, increase; −, decrease.

## Additional Information and Declarations

### Field Study Permissions

The following information was supplied relating to field study approvals (i.e., approving body and any reference numbers): The Western Australia, Department of Environment and Conservation (no. BB 002563).

### Data Availability

The following information was supplied regarding data availability: The data is presented in Tables S1–S3.
