# Supplementary material for: Extra-pair paternity in the long-tailed finch Poephila acuticauda
Source: PeerJ. 2016 Jan 5;4:e1550. doi: 10.7717/peerj.1550 (PMC4715429; doi:10.7717/peerj.1550)
Supplement: Table S1 — Data on the incidence of extra-pair offspring in 101 nests across the three years sorted by female ID and date order of nests (EPO, extra-pair offspring; WPO, within-pair offspring; Prop EP, proportion of offspring that are sired by extra-pair male). [file peerj-04-1550-s001.docx]

**Table S1** Data on the incidence of extra-pair offspring in 101 nests across the three years sorted by female ID and date order of nests (EPO - extra-pair offspring; WPO – within-pair offspring; Prop EP – proportion of offspring that are sired by extra-pair male)

| Nest ID | Year | Female ID | Male ID | EPO | WPO | Prop EP |
| --- | --- | --- | --- | --- | --- | --- |
| F36 | 2009 | 44878 | 61320 | 0 | 3 | 0.0 |
| 7 | 2008 | 44893 | 61111 | 2 | 1 | 0.7 |
| F17 | 2009 | 44893 | 61143 | 4 | 0 | 1.0 |
| F51 | 2009 | 44893 | 61157 | 0 | 3 | 0.0 |
| F67 | 2009 | 44893 | 61157 | 0 | 5 | 0.0 |
| F13 | 2009 | 44895 | 44884 | 0 | 2 | 0.0 |
| 9 | 2010 | 45492 | 61757 | 0 | 3 | 0.0 |
| 3 | 2010 | 45494 | 69902 | 2 | 1 | 0.7 |
| F05 | 2009 | 61117 | 61118 | 1 | 4 | 0.2 |
| F33 | 2009 | 61117 | 61118 | 0 | 4 | 0.0 |
| 17 | 2008 | 61126 | 61351 | 1 | 2 | 0.3 |
| 27 | 2008 | 61126 | 61351 | 0 | 3 | 0.0 |
| F02 | 2009 | 61126 | 61351 | 1 | 3 | 0.3 |
| F30 | 2009 | 61126 | 61351 | 0 | 2 | 0.0 |
| F59 | 2009 | 61126 | 61351 | 0 | 3 | 0.0 |
| 25 | 2008 | 61127 | 61109 | 0 | 4 | 0.0 |
| F01 | 2009 | 61127 | 61109 | 0 | 5 | 0.0 |
| 16 | 2008 | 61129 | 61390 | 0 | 4 | 0.0 |
| 13 | 2008 | 61141 | 61143 | 0 | 5 | 0.0 |
| 12 | 2008 | 61142 | 61131 | 0 | 3 | 0.0 |
| F40 | 2009 | 61142 | 61131 | 1 | 2 | 0.3 |
| F69 | 2009 | 61142 | 61131 | 1 | 3 | 0.3 |
| F03 | 2009 | 61170 | 61108 | 0 | 4 | 0.0 |
| F42 | 2009 | 61175 | 61329 | 0 | 4 | 0.0 |
| F65 | 2009 | 61175 | 61329 | 3 | 1 | 0.8 |
| 20 | 2008 | 61180 | 61146 | 0 | 3 | 0.0 |
| 24 | 2008 | 61180 | 61161 | 2 | 2 | 0.5 |
| F18 | 2009 | 61180 | 61161 | 0 | 5 | 0.0 |
| 3 | 2008 | 61183 | 61155 | 0 | 4 | 0.0 |
| F15 | 2009 | 61185 | 61184 | 0 | 5 | 0.0 |
| F46 | 2009 | 61189 | 61772 | 0 | 3 | 0.0 |
| F73 | 2009 | 61189 | 61772 | 0 | 4 | 0.0 |
| 22 | 2008 | 61195 | 61302 | 0 | 5 | 0.0 |
| F52 | 2009 | 61195 | 61302 | 2 | 1 | 0.7 |
| 9 | 2008 | 61196 | 61317 | 0 | 6 | 0.0 |
| 21 | 2008 | 61327 | 61326 | 0 | 5 | 0.0 |
| F38 | 2009 | 61327 | 61326 | 2 | 4 | 0.3 |
| 4 | 2008 | 61338 | 61316 | 0 | 2 | 0.0 |
| 5 | 2008 | 61338 | 61316 | 0 | 4 | 0.0 |
| 8 | 2008 | 61353 | 61348 | 0 | 5 | 0.0 |
| 2 | 2008 | 61361 | 61174 | 2 | 2 | 0.5 |
| 1 | 2008 | 61368 | 44885 | 0 | 4 | 0.0 |
| F25 | 2009 | 61368 | 44885 | 0 | 3 | 0.0 |
| F20 | 2009 | 61369 | 69558 | 0 | 4 | 0.0 |
| F56 | 2009 | 61369 | 69558 | 0 | 3 | 0.0 |
| F23 | 2009 | 61374 | 61566 | 0 | 5 | 0.0 |
| 11 | 2010 | 61374 | 61109 | 3 | 2 | 0.6 |
| 18 | 2010 | 61374 | 61109 | 0 | 4 | 0.0 |
| 14 | 2008 | 61389 | 61521 | 0 | 4 | 0.0 |
| 11 | 2008 | 61396 | 61537 | 0 | 3 | 0.0 |
| 6 | 2008 | 61399 | 61395 | 0 | 3 | 0.0 |
| 19 | 2008 | 61526 | 61533 | 1 | 4 | 0.2 |
| 15 | 2008 | 61538 | 61527 | 2 | 1 | 0.7 |
| F24 | 2009 | 61538 | 61527 | 0 | 4 | 0.0 |
| 10 | 2008 | 61552 | 61551 | 0 | 2 | 0.0 |
| F43 | 2009 | 61553 | 69579 | 0 | 5 | 0.0 |
| F70 | 2009 | 61553 | 69579 | 0 | 4 | 0.0 |
| 2 | 2010 | 61553 | 69579 | 0 | 2 | 0.0 |
| F09 | 2009 | 61583 | 61332 | 0 | 5 | 0.0 |
| F04 | 2009 | 61588 | 61144 | 0 | 5 | 0.0 |
| F32 | 2009 | 61706 | 61132 | 0 | 3 | 0.0 |
| F50 | 2009 | 61739 | 61138 | 3 | 1 | 0.8 |
| F19 | 2009 | 61794 | 45438 | 0 | 5 | 0.0 |
| F66 | 2009 | 61794 | 45438 | 0 | 4 | 0.0 |
| 10 | 2010 | 61847 | 61351 | 0 | 2 | 0.0 |
| F44 | 2009 | 61863 | 61384 | 1 | 1 | 0.5 |
| F07 | 2009 | 61879 | 61855 | 0 | 7 | 0.0 |
| F34 | 2009 | 61879 | 61855 | 0 | 5 | 0.0 |
| F64 | 2009 | 61879 | 61855 | 0 | 4 | 0.0 |
| 17 | 2010 | 61879 | 61855 | 0 | 5 | 0.0 |
| F11 | 2009 | 61894 | 61147 | 0 | 5 | 0.0 |
| F63 | 2009 | 61894 | 61147 | 0 | 4 | 0.0 |
| 16 | 2010 | 61894 | 61388 | 1 | 3 | 0.3 |
| F57 | 2009 | 61925 | 69902 | 1 | 3 | 0.3 |
| F26 | 2009 | 61933 | 61345 | 2 | 2 | 0.5 |
| F55 | 2009 | 61933 | 61345 | 0 | 5 | 0.0 |
| 5 | 2010 | 69111 | 61316 | 0 | 4 | 0.0 |
| 8 | 2010 | 69130 | 69171 | 0 | 5 | 0.0 |
| 15 | 2010 | 69162 | 61851 | 3 | 2 | 0.6 |
| F14 | 2009 | 69508 | 61315 | 0 | 4 | 0.0 |
| F72 | 2009 | 69508 | 61315 | 2 | 1 | 0.7 |
| 1 | 2010 | 69508 | 61331 | 0 | 4 | 0.0 |
| 4 | 2010 | 69528 | 61302 | 0 | 3 | 0.0 |
| F16 | 2009 | 69534 | 61321 | 2 | 1 | 0.7 |
| F49 | 2009 | 69534 | 61321 | 0 | 2 | 0.0 |
| F21 | 2009 | 69535 | 61137 | 0 | 3 | 0.0 |
| F62 | 2009 | 69535 | 61161 | 0 | 4 | 0.0 |
| F06 | 2009 | 69548 | 61316 | 0 | 6 | 0.0 |
| F27 | 2009 | 69548 | 61316 | 3 | 2 | 0.6 |
| F61 | 2009 | 69548 | 61316 | 0 | 4 | 0.0 |
| F37 | 2009 | 69701 | 61128 | 0 | 4 | 0.0 |
| F47 | 2009 | 69701 | 61128 | 2 | 1 | 0.7 |
| 7 | 2010 | 69701 | 61128 | 0 | 5 | 0.0 |
| F28 | 2009 | 69702 | 61531 | 0 | 5 | 0.0 |
| F71 | 2009 | 69702 | 61130 | 0 | 3 | 0.0 |
| F35 | 2009 | 69727 | 69596 | 0 | 3 | 0.0 |
| F29 | 2009 | 69771 | 61757 | 0 | 3 | 0.0 |
| F45 | 2009 | 69771 | 61757 | 0 | 3 | 0.0 |
| F53 | 2009 | 69793 | 61701 | 0 | 4 | 0.0 |
| 14 | 2010 | 69793 | 61701 | 0 | 2 | 0.0 |
| F54 | 2009 | 69901 | 61112 | 0 | 3 | 0.0 |
