# Supplementary material for: Extra-pair paternity in the long-tailed finch Poephila acuticauda
Source: PeerJ. 2016 Jan 5;4:e1550. doi: 10.7717/peerj.1550 (PMC4715429; doi:10.7717/peerj.1550)
Supplement: Table S2 — The proportion of extra-pair offspring in the successive broods in the (A) females and (B) males that bred multiple times within the same year (broods arranged in date order). Signs denote the direction of change over time: n/c, no change; +, increase; −, decrease. [file peerj-04-1550-s002.docx]

Table S2 The proportion of extra-pair offspring in the successive broods in the a) females and b) males that bred multiple times within the same year (broods arranged in date order). Signs denote the direction of change over time: n/c, no change; +, increase; -, decrease

a)

| Female ID | Year | Brood 1 | Brood 2 | Brood 3 | Direction |
| --- | --- | --- | --- | --- | --- |
| 61189 | 2009 | 0 | 0 |  | n/c |
| 61338 | 2008 | 0 | 0 |  | n/c |
| 61369 | 2009 | 0 | 0 |  | n/c |
| 61553 | 2009 | 0 | 0 |  | n/c |
| 61794 | 2009 | 0 | 0 |  | n/c |
| 61879 | 2009 | 0 | 0 | 0 | n/c |
| 61894 | 2009 | 0 | 0 |  | n/c |
| 69535 | 2009 | 0 | 0 |  | n/c |
| 69702 | 2009 | 0 | 0 |  | n/c |
| 69771 | 2009 | 0 | 0 |  | n/c |
| 61180 | 2008 | 0 | 0.50 |  | + |
| 69548 | 2009 | 0 | 0.60 | 0 | + and - |
| 69508 | 2009 | 0 | 0.67 |  | + |
| 69701 | 2009 | 0 | 0.67 |  | + |
| 61175 | 2009 | 0 | 0.75 |  | + |
| 61117 | 2009 | 0.20 | 0 |  | - |
| 61126 | 2009 | 0.25 | 0 | 0 | - |
| 61126 | 2008 | 0.33 | 0.00 |  | - |
| 61142 | 2009 | 0.33 | 0.25 |  | - |
| 61933 | 2009 | 0.50 | 0 |  | - |
| 61374 | 2010 | 0.60 | 0 |  | - |
| 69534 | 2009 | 0.67 | 0 |  | - |
| 44893 | 2009 | 1.00 | 0 | 0 | - |

b)

| Male ID | Year | Brood 1 | Brood 2 | Brood 3 | Direction |
| --- | --- | --- | --- | --- | --- |
| 61316 | 2008 | 0 | 0 |  | n/c |
| 61157 | 2009 | 0 | 0 |  | n/c |
| 61329 | 2009 | 0 | 0.75 |  | + |
| 61772 | 2009 | 0 | 0 |  | n/c |
| 69558 | 2009 | 0 | 0 |  | n/c |
| 69579 | 2009 | 0 | 0 |  | n/c |
| 45438 | 2009 | 0 | 0 |  | n/c |
| 61855 | 2009 | 0 | 0 | 0 | n/c |
| 61147 | 2009 | 0 | 0 |  | n/c |
| 61315 | 2009 | 0 | 0.67 |  | + |
| 61316 | 2009 | 0 | 0.60 | 0 | + and - |
| 61128 | 2009 | 0 | 0.67 |  | + |
| 61757 | 2009 | 0 | 0 |  | n/c |
| 61118 | 2009 | 0.20 | 0 |  | - |
| 61351 | 2009 | 0.25 | 0 | 0 | - |
| 61351 | 2008 | 0.33 | 0 |  | - |
| 61131 | 2009 | 0.33 | 0.25 |  | - |
| 61345 | 2009 | 0.50 | 0 |  | - |
| 61109 | 2010 | 0.60 | 0 |  | - |
| 61321 | 2009 | 0.67 | 0 |  | - |
