# Supplementary material for: Extra-pair paternity in the long-tailed finch Poephila acuticauda
Source: PeerJ. 2016 Jan 5;4:e1550. doi: 10.7717/peerj.1550 (PMC4715429; doi:10.7717/peerj.1550)
Supplement: Table S3 — The proportion of extra-pair offspring in the successive broods in the (A) females and (B) males that bred across two different years (the values are from their broods that are last in year 1 and earliest in year 2). Signs denote the direction of change over time: n/c, no change; +, increase; −, decrease. [file peerj-04-1550-s003.docx]

Table S3 The proportion of extra-pair offspring in the successive broods in the a) females and b) males that bred across two different years (the values are from their broods that are last in year 1 and earliest in year 2). Signs denote the direction of change over time: n/c, no change; +, increase; -, decrease

a)

| Female ID | EPP year 1 | EPP year 2 | Direction |
| --- | --- | --- | --- |
| 61127 | 0 | 0 | n/c |
| 61368 | 0 | 0 | n/c |
| 61553 | 0 | 0 | n/c |
| 61879 | 0 | 0 | n/c |
| 69793 | 0 | 0 | n/c |
| 44893 | 0.67 | 1.00 | + |
| 61126 | 0 | 0.25 | + |
| 61142 | 0 | 0.33 | + |
| 61195 | 0 | 0.67 | + |
| 61327 | 0 | 0.33 | + |
| 61374 | 0 | 0.60 | + |
| 61894 | 0 | 0.25 | + |
| 61538 | 0.67 | 0 | - |
| 69701 | 0.67 | 0 | - |
| 61180 | 0.50 | 0 | - |
| 69508 | 0.67 | 0 | - |

b)

| Male ID | EPP year 1 | EPP year 2 | Direction |  |
| --- | --- | --- | --- | --- |
| 61109 | 0 | 0 | n/c |  |
| 44885 | 0 | 0 | n/c |  |
| 69579 | 0 | 0 | n/c |  |
| 61855 | 0 | 0 | n/c |  |
| 61701 | 0 | 0 | n/c |  |
| 61351 | 0 | 0.25 | + |  |
| 61131 | 0 | 0.33 | + |  |
| 61302 | 0 | 0.67 | + |  |
| 61326 | 0 | 0.33 | + |  |
| 61527 | 0.67 | 0 | - |  |
| 61161 | 0.50 | 0 | - |  |
| 61128 | 0.67 | 0 | - |  |
